# Diazirine-Functionalized Polyurethane Crosslinkers for Isocyanate-Free Curing of Polyol-Based Coatings

**DOI:** 10.1021/acsapm.4c00266

**Published:** 2024-03-05

**Authors:** Felix J. de Zwart, Lukas A. Wolzak, Keimpe J van den Berg, Miranda J. Baran, Jeremy E. Wulff, Jitte Flapper, Bas de Bruin

**Affiliations:** † Homogeneous, Supramolecular and Bio-Inspired Catalysis Group, van ’t Hoff Institute for Molecular Sciences (HIMS), University of Amsterdam, 1098 XH Amsterdam, The Netherlands; ‡ Akzo Nobel Car Refinishes B.V., 2171 AJ Sassenheim, The Netherlands; § XLYNX Materials, Inc., Victoria, British Columbia V8P 5C2, Canada; ∥ Department of Chemistry, University of Victoria, Victoria, British Columbia V8W 2Y2, Canada; ⊥ Akzo Nobel Decorative Coatings B.V., 2171 AJ Sassenheim, The Netherlands

**Keywords:** carbenes, polyurethanes, coating materials, crosslinking, mechanical properties

## Abstract

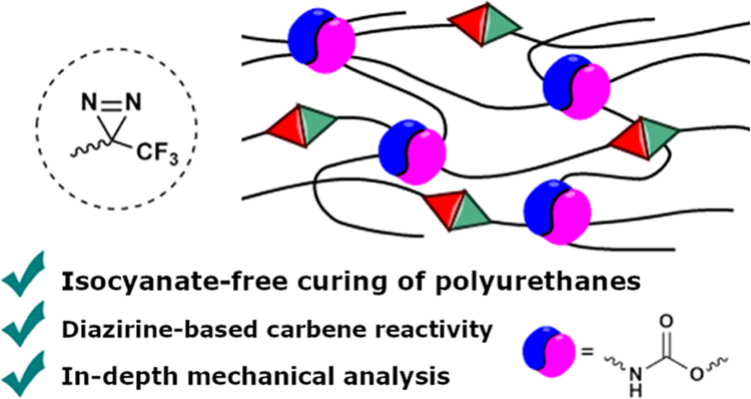

Polyurethane coatings have strong material properties due to the hydrogen bonding inherent to the urethane groups. However, installing this urethane moiety usually requires curing through difficult-to-handle isocyanates. In this work, we show the development of a polyurethane-based crosslinker that can be used to formulate a one-component polyurethane coating with material properties similar to those of isocyanate-based polyurethane coatings. To achieve this, we used diazirine functionalities that generate carbenes upon heating, which react with alcohol functionalities in a polyol to generate a crosslinked network with a high storage modulus.

## Introduction

**Figure 1:**
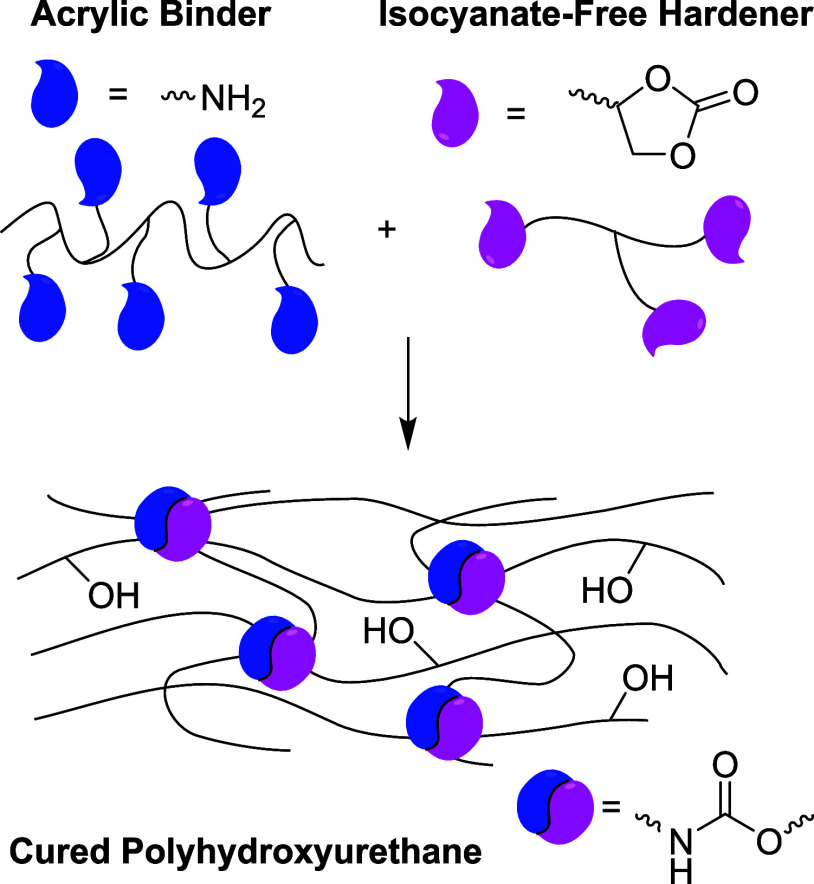
Carbonate-based isocyanate-free routes toward polyurethanes constructing urethane moieties in the final polymerizing step, leading to polyhydroxyurethanes.

Polyurethanes (PURs) and polyurea (PU) materials have garnered substantial interest due to their versatile properties and wide-ranging applications across industries such as coatings, adhesives, foams, and textiles. Isocyanates have long been the linchpin of polyurethane coatings, conferring excellent mechanical properties, chemical resistance, and adhesion. The inherent strength of these materials is rooted in the intermolecular interactions facilitated by hydrogen bonds, allowing them to serve as structural components and functional coatings in a variety of contexts.^1^ Notably, the exceptional material properties and chemical resistance of polyurethane materials have secured a pivotal position in the landscape of polymer materials. However, the reliance on isocyanate-based curing agents has sparked concerns due to their potential health hazards and the resulting legislative pressure. In response, innovative approaches are being explored to replace isocyanate-based systems with safer and more sustainable alternatives.^2^ The desirable properties of polyurethanes are an inherent result of the unique primary and secondary structures of the urethane linkage. The linkage itself is naturally resistant to chemicals and solvents, and its self-hydrogen-bonding properties induce the organization of polymeric chains into microdomains, which improve the bulk properties of the material.^3^ Most isocyanate-free routes to PUR-like coatings are based on species such as carbonates, urethanes, and ureas.^4,5^ In these aminolysis and transcarbamoylation reactions, polyurethanes are synthesized by generating the final urethane linkage in the polymerization step (Figure 1).^6^ These isocyanate-free routes require catalysts and high temperatures, which can lead to side reactions forming urea, uretdione, isocyanurate, and allophanate linkages. Furthermore, carbonate aminolysis leads to polyhydroxyurethanes (PHUs), which contain stronger hydrogen bonding networks due to the additional hydroxyl groups present.^7,8^ These stronger hydrogen bonding networks limit the mobility of species during the reaction, thereby limiting the conversion during polymerization, which leads to reduced final molar masses of the PHUs.^9,10^

**Figure 2:**
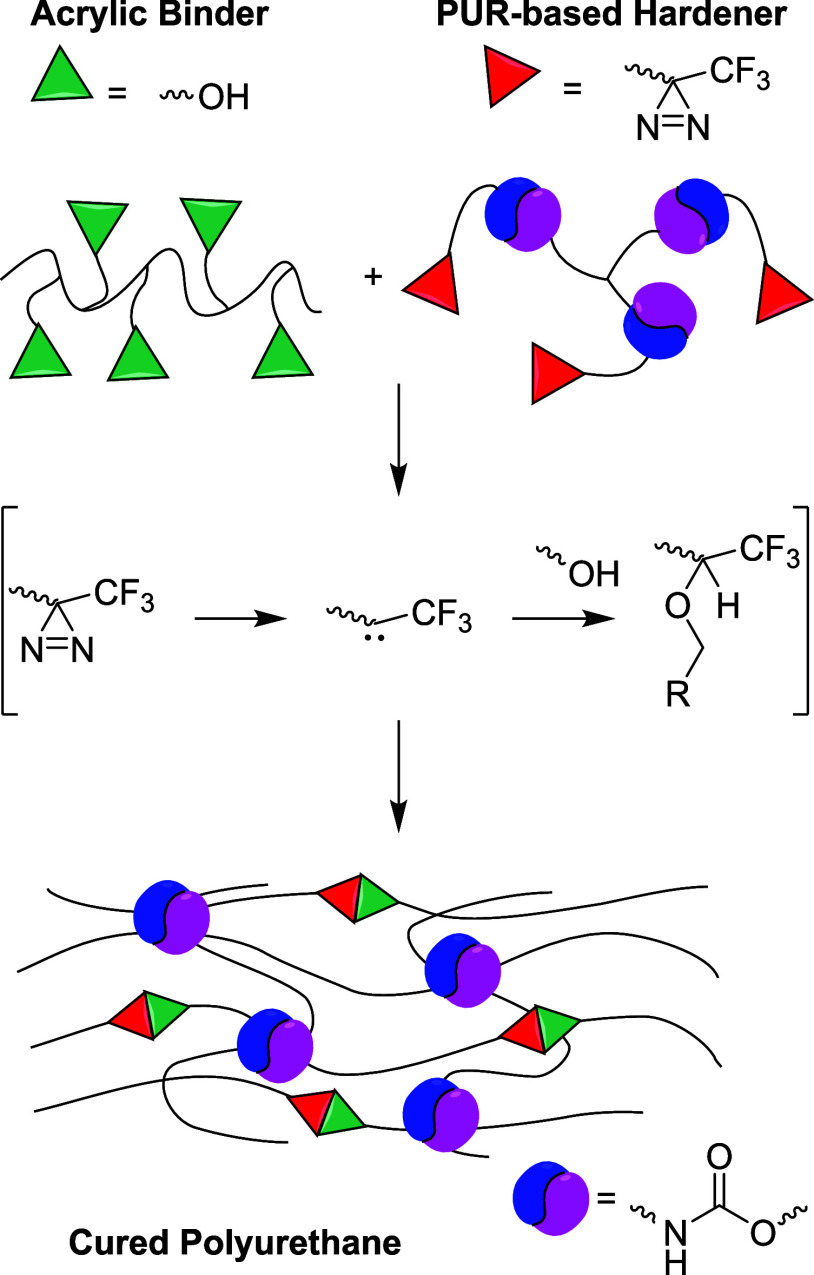
Isocyanate-free routes toward polyurethanes utilizing alternative polymerizing reactions for final curing, displaying the envisioned strategy of carbene insertion.

An alternative route toward isocyanate-free polyurethanes utilizes prepolymers that contain the crucial urethane linkages installed before the final polymerization (Figure 2). By functionalizing polyurethane with acrylate moieties, the final curing step can be performed through acrylate polymerization.^11^ Another elegant application utilizes click chemistry to cure polyurethane coatings. By incorporating azide functionalities in an acrylic binder and alkyne functionalities in polyurethane, the group of Storey synthesized coatings that can be cured through copper click chemistry.^12,13^ Inspired by this, we wondered whether it would be possible to achieve isocyanate-free curing of a polyurethane coating by functionalizing only the polyurethane component. Most commonly, two-component polyurethanes consist of a polyol binder and a polyisocyanate hardener. We aimed to functionalize the latter with a reactive yet conveniently storable, easily manageable, and less toxic moiety that undergoes traceless crosslinking with the polyol.^14^

Carbene-generating crosslinkers have had a large commercial impact on materials chemistry, finding applications as fabric strengtheners,^15^ adhesives,^16^ and in mixed plastic recycling.^17^ In particular, diazirine compounds have shown high efficiency in crosslinking polymers bereft of specific functionalities (e.g., poly(dimethylsiloxane)^18^) through C–H insertion, and functional polymers (e.g., containing hydroxyl and thiol functionalities^19^) through X–H insertion. A recent report on structure–function relationships in aryl diazirines shows the usage of *para*-methoxy-functionalized aryl diazirines to selectively achieve O–H insertion.^20^ Based on this, we present the development of a diazirine-based polyurethane crosslinker that utilizes carbene O–H insertion as an alternative isocyanate-free curing for PUR coating.

## Results and Discussion

**Figure 3:**
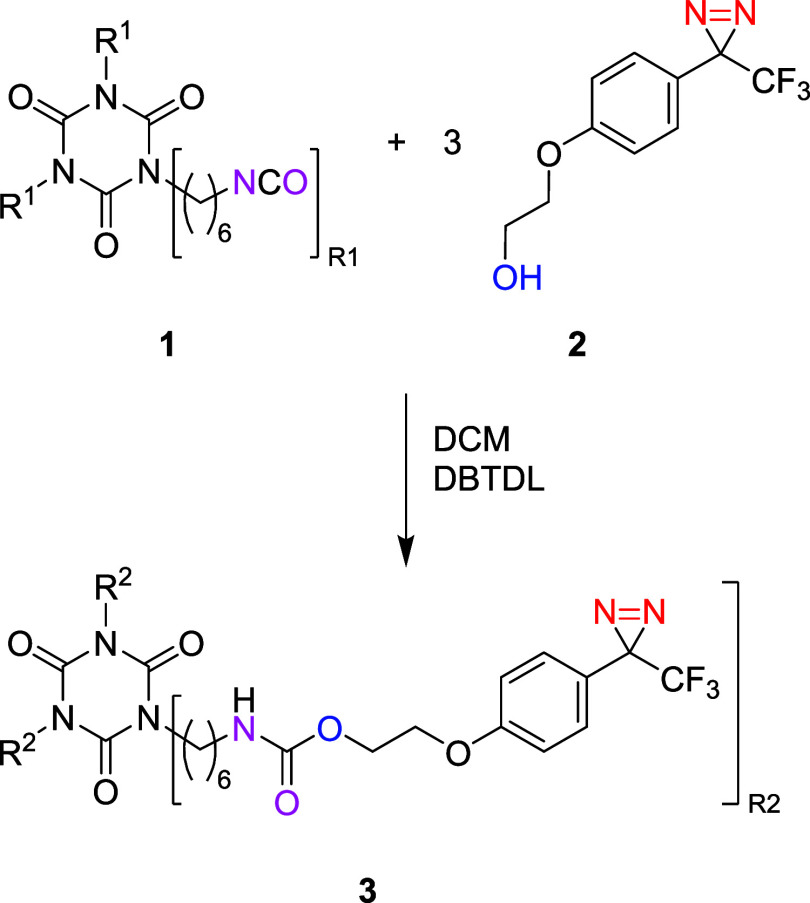
Synthesis of PUR-based crosslinker **3** using the hexamethylenediisocyanate trimer and glycol-functionalized diazirine (DBTDL = dibutyltin dilaurate).

As a polyurethane hardener, we developed a crosslinker, synthesized by the chemical modification of an oligomeric mixture of hexamethylenediisocyanate, which consists mainly of the isocyanurate hexamethylenediisocyanate trimer **1** (Figure 3). This compound is frequently used as an isocyanate crosslinker and is formulated as an oligomeric mixture to not contain any volatile isocyanate. The isocyanate groups in this crosslinker can be easily functionalized with an alcohol moiety. After tin-catalyzed isocyanate alcoholysis with ethylene glycol-functionalized diazirine **2**, the PUR-based crosslinker **3** is obtained (Figure 3). The diazirine functionality was selected over a similar diazo functionality based on two advantages: diazirines are not as colored, whereas aryl/ester diazos absorb in the visible range, and diazirines are less polar, which helps with solubility in apolar-solvent-based acrylics.

ATR-FTIR spectroscopy confirmed the full conversion of the isocyanate moieties through the disappearance of the band at 2274 cm^–1^ (Figure S5). Through ^1^H- and HRMS, we confirmed the main species to be the isocyanurate-based trimer with three aryltrifluoromethyldiazirine arms attached. ^1^H NMR spectroscopy indicated the presence of a minor (<5%) species with additional signals in the aromatic (7.3 ppm) region, which could originate from allophanate or biuret-type species generated during isocyanate alcoholysis (Figure S1).^21^ Most importantly, ^13^C NMR spectra confirmed the aryltrifluoromethyldiazirine moiety to be intact through the presence of a quartet at 28 ppm (Figure S2).^22^ The crosslinker was then subjected to thermochemical analysis through differential scanning calorimetry (DSC).^23^ With a measured enthalpy of decomposition Δ*H*_D_ of 338 J/g and a *T*_onset_ of 88 °C, this compound was found unlikely to be explosive (Figure S6). Moreover, the *T*_onset_ and the molar enthalpy of decomposition per diazirine group (140 kJ/mol) are identical to those of 3-(4-methoxyphenyl)-3-(trifluoromethyl)-3*H*-diazirine, indicating that the electronic properties of the diazirine group are dominated by the *para*-alkoxy substituent on the arene.^20^ The choice of the *para*-alkoxy substituent is of great importance in the envisioned application, as electron-rich diazirines yield singlet carbenes, which favor O–H insertion relative to triplet carbenes.^24^ Furthermore, even in the presence of an acrylic binder, the crosslinker shows no rapid decomposition until 80 °C, indicating that this PUR-diazirine acrylic composite could have a long shelf life as a one-component coating.

**Figure 4:**
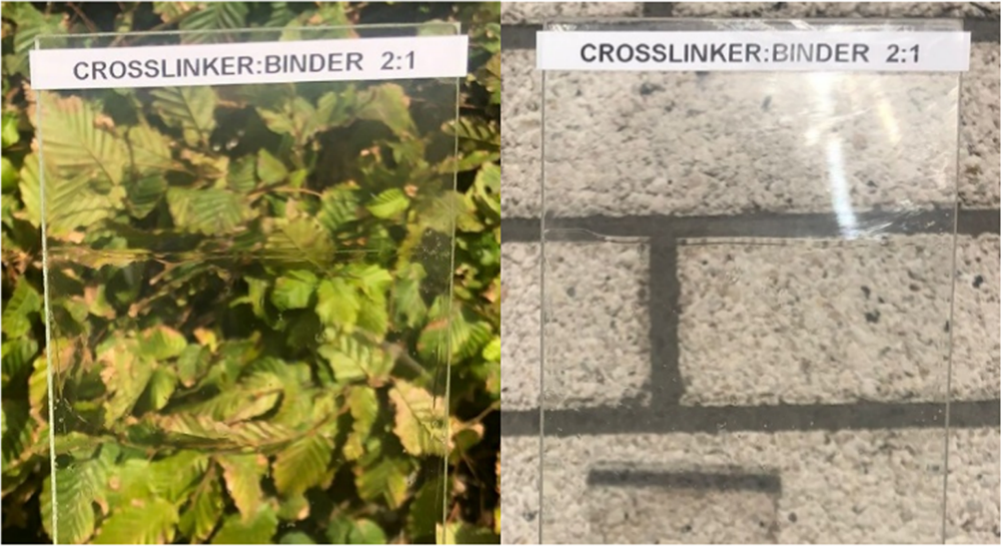
Images displaying optically clear coatings (approximately 40 μM final thickness) containing a 2:1 ratio of crosslinker **3** to binder on a glass panel.

To evaluate the performance of the PUR-diazirine crosslinker, it was tested in the thermal crosslinking of the APO binder by adding increasing amounts of the crosslinker up to a 2:1 molar ratio of diazirine (crosslinker functionality) to hydroxyl (binder functionality). The acrylic polyol (APO) binder used in this work is a butyl (meth)acrylate-based binder with (hydroxyethyl)methacrylate as its main functionality (hydroxyl equivalent weight 410 g/mol), synthesized according to the literature.^25^ The crosslinker was activated thermally by heating the coatings to 110 °C overnight. This provided clear coatings on glass panels, even at the highest loading of the crosslinker (Figure 4). The hardness of the films was evaluated by the Persoz pendulum testing and compared to a traditional polyurethane coating based on the same APO binder and polyisocyanate **1** as the hardener. While at a 1:1 ratio, the traditional crosslinker **1** outperforms the diazirine crosslinker **3** with a hardness of 342 against 251 oscillations (Table 1), the hardness of coatings cured at a 2:1 ratio is equally high for both crosslinkers. This indicates that at a high enough loading, the developed crosslinker **3** provides coatings that are just as hard as those based on traditional isocyanate technology.

**Table 1:** Persoz Hardness (Average of Duplo) of Cured Coatings Based on Crosslinkers **1** and **3**

binder	crosslinker	mol ratio (NCO or N_2_)/OH	Persoz (oscillations)
APO	none	-	95
APO	isocyanate **1**	1:1	342
APO	isocyanate **1**	2:1	354
APO	diazirine **3**	1:1	251
APO	diazirine **3**	2:1	364

**Figure 5:**
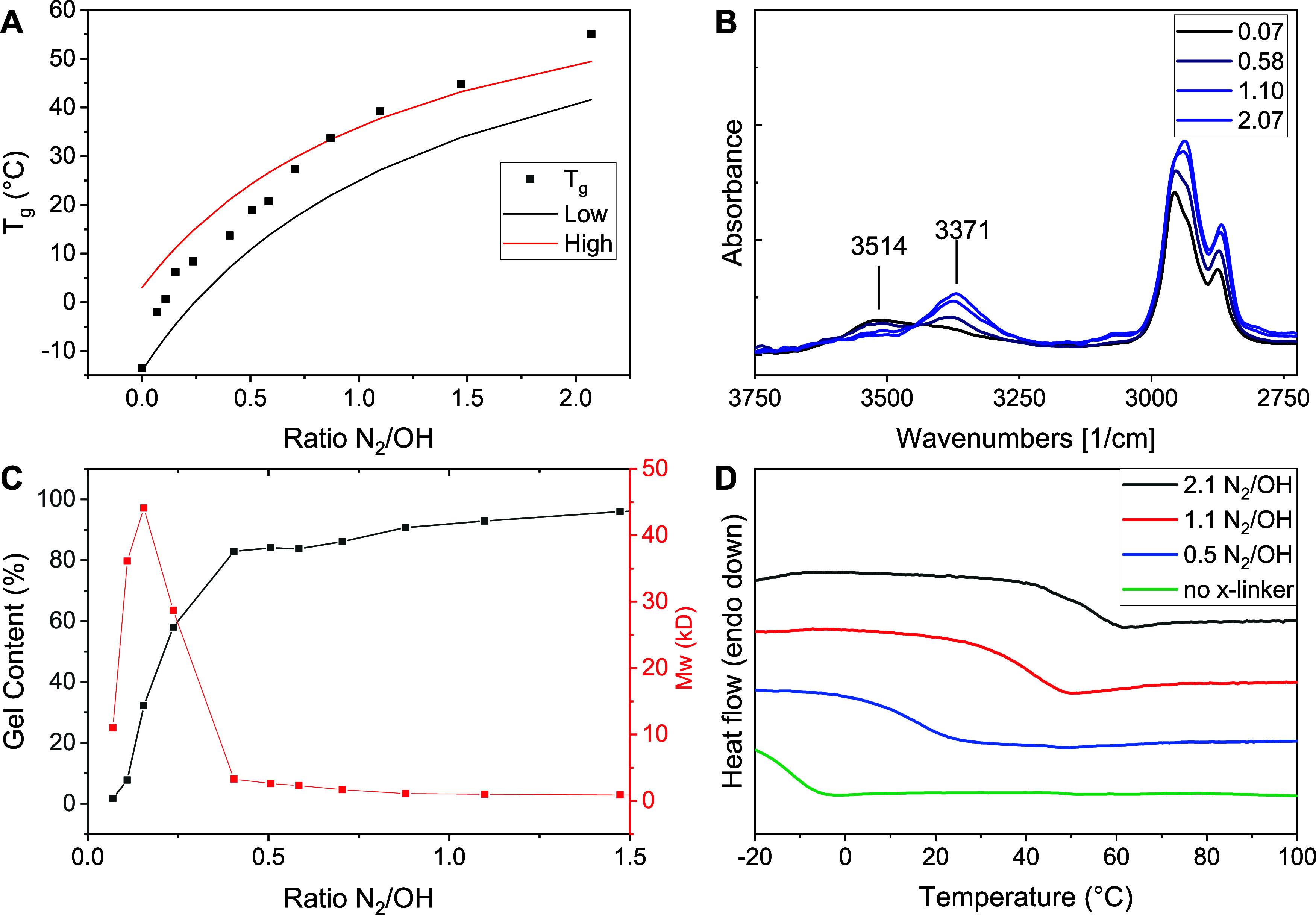
Characterization of APO-based coatings with increasing amounts of crosslinker **3**. (A) Glass transition temperature as measured by DSC. (B) ATR-FTIR spectra displaying a change in the intensity of bands at 3514 and 3371 cm^–1^. (C) Gel content of coating, and molecular weight of soluble fraction as measured by GPC. (D) Representative DSC traces displaying an increase in *T*_g_.

Using DSC, we evaluated the change in the *T*_g_ of the cured coating (Figure 5A). Adding increasing amounts of the crosslinker leads to a sharp increase in the *T*_g_ of the polymer up to 55 °C. Infrared spectroscopy revealed a steady decrease in the O–H vibration band at 3514 cm^–1^ and an increase in the N–H vibration band at 3371 cm^–1^, the latter stemming from the increasing amount of urethane moieties in the composite (Figure 5B). The intensity of the O–H band decreases beyond a crosslinker ratio of 1:1 N_2_/OH, indicating that the crosslinking reaction is not fully selective for O–H insertion. The molecular weight of the soluble contents of the coating peaks at a ratio of 0.16 N_2_/OH and then decreases rapidly, accompanied by a rapid increase in the gel content (Figure 5C), indicating the formation of large insoluble networks.

When using a pure PUR-diazirine crosslinker without the addition of APO, we observed a *T*_g,PUR_ of 81 °C. In the absence of the solvent, the APO, which starts at a low molecular weight, has a *T*_g,APO_ of −14 °C, and the theoretical Fox *T*_g,APO_ of this acrylic is known to be 3 °C at an infinite molecular weight.^26^ Using these values and Fox eq 1, where *w* is the weight fraction, we can estimate the expected *T*_g_ of the coating as follows
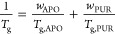
1The expected *T*_g_ of the composite can be estimated based on two situations: (1) a lower limit in which the crosslinker purely reacts with itself, and under these crosslinking conditions, no increase in the molecular weight of the APO occurs (Figure 5A, low); (2) an upper limit in which the crosslinker purely reacts with itself, and under these crosslinking conditions, the APO also forms an infinite network (Figure 5A, high). As can be seen in Figure 5A, the observed *T*_g_ of the composite breaks the upper limit above a ratio of 1:1 N_2_/OH, indicating chemical modification of the polymer composite.^27^ This is in line with the expected O–H insertion and further confirms the formation of crosslinking between PUR and APO.

To assess whether these coatings truly reflect the material properties of commercial polyurethanes, we performed a dynamical mechanical thermal analysis (DMTA) to assess the thermomechanical behavior of the coatings with increasing amounts of the crosslinker at N_2_/OH ratios of 2:1 and 1:1. The storage modulus (*E*′), loss modulus (*E*″), and tan δ values as a function of temperature are plotted in Figures S9 and S10, and the results are summarized in Table 2.^28^ The coating properties remain stable until 180 °C, as evidenced by the plateau of the storage modulus. For increased amounts of the crosslinker, a higher storage modulus is evident in the rubbery region, and a broadening and shift toward higher temperatures is observed for the tan δ peak. With increasing amounts of the crosslinker, the storage modulus at room temperature increases from 1.4 to 2.0 GPa, which is in line with traditionally cured polyurethane coatings.^29^ The *T*_g_ values acquired from the DMTA measurements are higher than the *T*_g_ obtained from DSC, which can be attributed to the frequency effect.^30^ We determined the *T*_g_ of the reference isocyanate coatings through DSC measurements and found this to be 56 °C at an equimolar loading of NCO to hydroxyl (Figure S11). This agrees with the Persoz hardness measurements, indicating that the material properties of traditionally cured isocyanate coatings are similar to those of the carbene technology developed herein. For a crosslinked polymer, the storage modulus value in the rubbery plateau region is correlated with the number of crosslinks in the polymer chain. As can be seen from the *E*′ (90 °C), the storage modulus of the coating containing a higher amount of the crosslinker is significantly higher. This storage modulus was then used to determine the crosslinking density based on the calculated molecular weight between crosslinks *M*_c_. This shows that an increase in the hardness is accompanied by an increase in the crosslinking density. From the DMTA analysis, it can clearly be concluded that an increasing amount of PUR-diazirine crosslinker leads to an improvement in the mechanical properties of the coating through crosslinking.

**Table 2:** Processed Data from the DMTA Analysis of Coatings Cured Using Crosslinker **3**^a^

ratio N_2_/OH	*E*′ (−20 °C) (MPa)	E′ (23 °C) (MPa)	*E*′ (90 °C) (MPa)	*T*_g_ (tan δ) (°C)	*T*_g_ (*E*″) (°C)	*T*_g_ (DSC) (°C)	*M*_c_ (g/mol)
1:1	2061	1356	3	48	32	35	3384
2:1	2262	2024	7	64	52	51	1354

a *E*′, storage modulus in MPa; *T*_g_ glass transition temperature in °C; *M*_c_ crosslink density in g/mol.

## Conclusions

We synthesized a diazirine-based crosslinker that contains urethane groups within the structure. Upon heating, this generates free carbenes that can be inserted into the O–H bonds of acrylic polyols. We show that by combining this crosslinker with a polyol, an isocyanate-free one-component coating can be formulated that retains the material properties of a traditional PUR coating, such as a high storage modulus. A higher crosslinking density of the final coating can be obtained by formulating an excess of the PUR crosslinker. This research paves the way for the use of free carbenes as an alternative curing method for isocyanate-free polyurethanes.

## Supporting Information Available

Synthesis and characterization of the crosslinker and coatings (PDF)
