# Supplementary material for: Diazirine-Functionalized Polyurethane Crosslinkers for Isocyanate-Free Curing of Polyol-Based Coatings
Source: ACS Appl Polym Mater. 2024 Mar 5;6(6):3517–22. doi: 10.1021/acsapm.4c00266 (PMC10964194; doi:10.1021/acsapm.4c00266)
Supplement: Supplementary file 1 — ap4c00266_si_001.pdf [file ap4c00266_si_001.pdf]

Supporting Information

Diazirine-Functionalized Polyurethane Crosslinkers for Isocyanate-Free Curing of Polyol-based Coatings

Felix J. de Zwart,[a]* Lukas A. Wolzak,[b] Keimpe J. van den Berg,[b] Miranda J. Baran,[c] Jeremy E. Wulff,[d] Jitte Flapper,[e] Bas de Bruin[a]*

[a] Homogeneous, Supramolecular and Bio-Inspired Catalysis Group, van 't Hoff Institute for Molecular Sciences (HIMS), University of Amsterdam, 1098 XH Amsterdam, The Netherlands

[b] Akzo Nobel Car Refinishes B.V., 2171 AJ Sassenheim, The Netherlands

[c] XLYNX Materials, Inc., Victoria, British Columbia V8P 5C2, Canada

[d] Department of Chemistry, University of Victoria, Victoria, British Columbia V8W 2Y2, Canada

[e] Akzo Nobel Decorative Coatings B.V., 2171 AJ Sassenheim, The Netherlands

*Corresponding Authors

E-mail: f.j.dezwart@uva.nl, b.debruin@uva.nl

1. General Considerations..3
2. Experimental Procedures...4
Thermal crosslinking procedure for GPC, gel content, DSC and IR...4
Thermal crosslinking procedure for DMTA...4
Synthesis of PUR-diazirine crosslinker **3** 5
3. Characterization of Crosslinker...6
4. Characterization of Coatings...9
References 11

1. General Considerations.

Chemicals and solvents were purchased from commercial suppliers and used as received. Compound **2** (2-(4-(3-(trifluoromethyl)-3*H*-diazirin-3-yl)phenoxy)ethan-1-ol) was synthesized according to literature.[1] Acrylic polyol (APO) was synthesized according to literature.[2,3] Briefly, this is a butyl (meth)acrylate-based binder with (hydroxyethyl)methacrylate as its main functionality. The hydroxyl equivalent weight of this APO was determined to be 410 g mol^{-1} (4.2 wt% based on solids). ^{1}H NMR spectra were acquired at room temperature on Bruker DRX 400 and 300 MHz instruments. NMR chemical shifts are reported in ppm and are referenced internally to the residual solvent peak of $CDCl_3$ (= 7.26 ppm). The glass transition temperature (T_g) of the polymers was determined by differential scanning calorimetry (DSC). IR spectra were recorded on a Bruker Alpha FTIR apparatus.

DSC measurements were performed on a Perkin Elmer Jade DSC. Samples were heated from 20 °C to 180 °C at a heating rate of 10°C/min followed by an isothermal step for 5 min. A cooling cycle to −20°C at a rate of 10°C/min was performed prior to a second heating run to 180°C at the same heating rate. The T_g was defined as the temperature of the midpoint of a heat capacity change on the second heating run. The Universal Analysis 2000 software was used for data acquisition.

SEC measurements were performed on a Shimadzu LC-20AD system with two PLgel 5 µm MIXED-C columns (Polymer Laboratories) in series and a Shimadzu RID-10A refractive index detector. CH_2Cl_2 was used as mobile phase at a flow rate of 1 mL/min and T = 35 °C. Polystyrene standards in the range of 760 – 1 880 000 g mol-1 (Sigma Aldrich) were used for calibration.

Dynamic thermal-mechanical analysis (DMTA) was performed on a TA Instruments RSA3 in a temperature range of −60 °C to 180 °C. Samples with dimensions 15 mm x 6 mm x 0.04 mm were tested with uniaxial stretching performed with a heating rate of 5 °C min^{-1}, frequency of 10 Hz and strain of 0.03%. The storage modulus (E') and the loss factor (tan δ) were recorded as a function of temperature. In order to estimate the apparent cross-linking density (M_c), the storage modulus in the rubbery plateau region (E'_R) was extracted from the graph. Equation (1) was used to calculate the M_c,[4,5] where M_c is the molecular weight between crosslinks, R is the gas constant, T is the absolute temperature.

Equation (1) $$M_c = \frac{3RT\rho}{E'_R}$$

2. Experimental Procedures.

Thermal crosslinking procedure for GPC, gel content, DSC and IR

In a typical procedure, acrylic polyol (50 mg, 75 wt% solids in n-butyl acetate, 0.091 mmol OH) was added to a preweighed 4 mL vial together with PUR-diazirine **3** (37.5 mg, 0.091 mmol N_2, 1:1 N_2/OH ratio). Thereafter, the samples were heated to 110 °C overnight. After cooling down to room temperature, a sample (2–5 mg) was taken for DSC and IR measurements. Thereafter, the vial was weighed again before adding DCM (4 mL) to the vial. After vortexing, the vial was centrifuged to settle all solids and the supernatant was removed to analyze the soluble contents by GPC. The leftover solids were dried in vacuo and weighed to determine the gel content of the coating.

Thermal crosslinking procedure for DMTA

In a typical procedure, crosslinker 1 (0.75 g, 1.8 mmol N_2) was added to acrylic polyol (1 g in butyl acetate, 0.75 g solids, 1.8 mmol N_2) together with ethyl acetate (approximately 100 µL) to achieve the desired viscosity for film formation. Using a drawbar, 90 µM thick films were cast onto polypropylene substrates which were heated to 110 °C for 16 hours in a well-ventilated oven. After cooling down to room temperature, the films were removed using a razor and dog-bone shapes were punched for DMTA measurements.

Synthesis of PUR-diazirine crosslinker **3**

To a flame-dried 20 mL Schlenk flask under nitrogen was added Tolonate HDT-LV **1** (1.46 g, 8.0 mmol NCO, 1 eq), one drop of dibutyl tin dilaurate and dry DCM (5 mL). The mixture was cooled to 0 °C, and 2-(4-(3-(trifluoromethyl)-3*H*-diazirin-3-yl)phenoxy)ethan-1-ol **2** (2.00 g, 8.1 mmol, 1.1 eq) in dry DCM (2 mL) was added dropwise. The cooling bath was removed afterwards, and the solution was stirred overnight.

At this point the acrylic polyol (1.64 g solids, 4 mmol OH) can directly be added to provide a stock solution of 2:1 N_2/OH, for which the equivalent weight of N_2 can be calculated using ^{19}F NMR against an internal standard (such as 3,4-dichlorofluorobenzene). This mixture can be further diluted using additional APO to the desired loading and can be cured without a difference in performance compared to the purified crosslinker.

To properly characterize the crosslinker, and evaluate the thermochemical properties, the mixture was purified by column chromatography (40% ethyl acetate in cyclohexane, R_f = 0.30) to provide the tritopic PUR-diazirine crosslinker **3** as a colorless oil (2.2 g, 1.77 mmol, 67% yield).

^{1}H NMR (300 MHz, Chloroform-d) δ 7.13 (d, J = 8.6 Hz, 6H), 6.90 (d, J = 8.9 Hz, 6H), 4.84 (s, 3H), 4.39 (t, J = 4.6 Hz, 6H), 4.13 (t, J = 4.7 Hz, 6H), 3.84 (t, 7.2 Hz, 6H), 3.15 (q, J = 6.7 Hz, 6H), 1.68 – 1.34 (m, 24H).

^{19}F NMR (282 MHz, Chloroform-d) δ -65.62.

^{13}C NMR (75 MHz, Chloroform-d) δ 159.72, 156.27, 149.10, 128.31, 122.34 (q, J = 274.7 Hz), 121.50, 115.07, 66.73, 62.99, 42.93, 41.03, 29.85, 28.33 (q, J = 40.4 Hz), 27.79, 26.36.

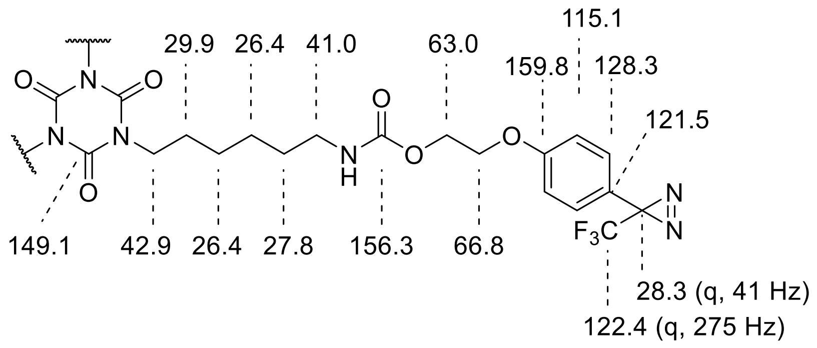

HRMS (ESI) m/z calculated $[M+Na]^+$: 1265.4437, found: 1265.4386, calculated $[M+NH_4]^+$: 1260.4883, found: 1260.4844.

3. Characterization of Crosslinker

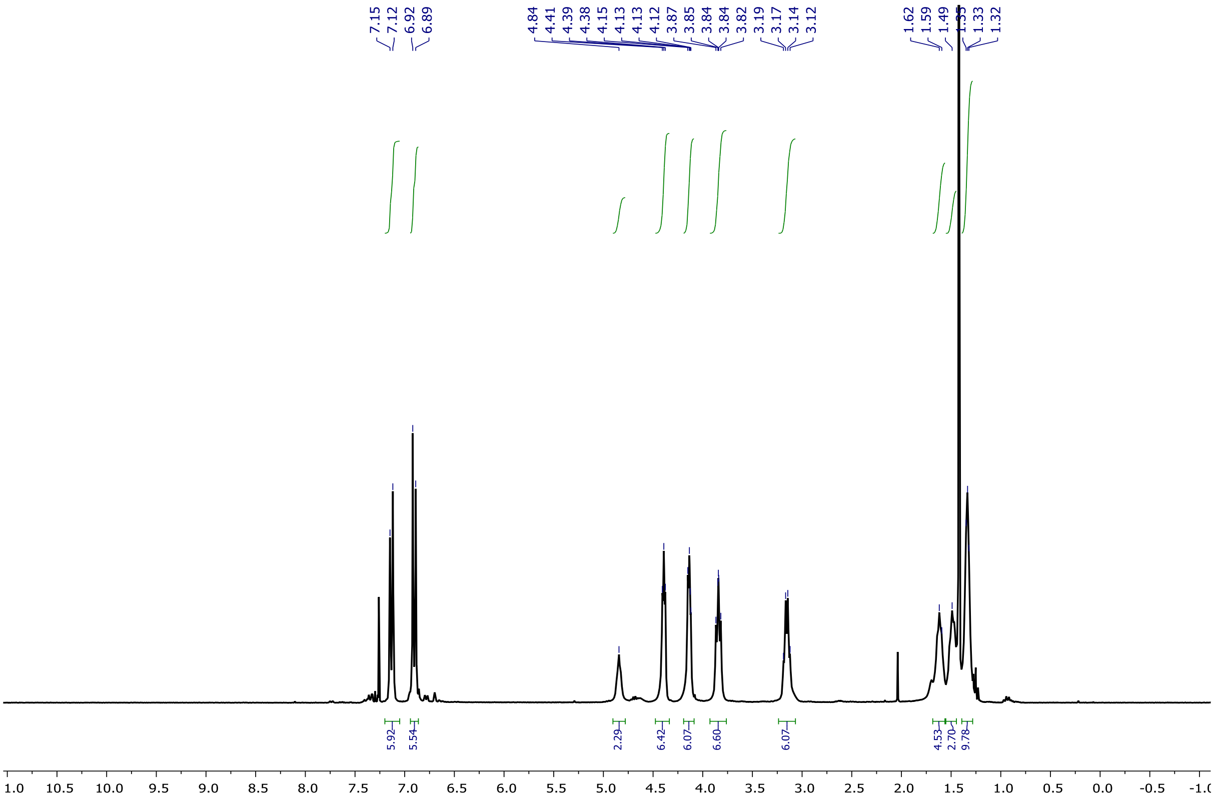

Figure S1. ^{1}H-NMR spectrum of PUR-diazirine crosslinker **3** in $CDCl_3$.

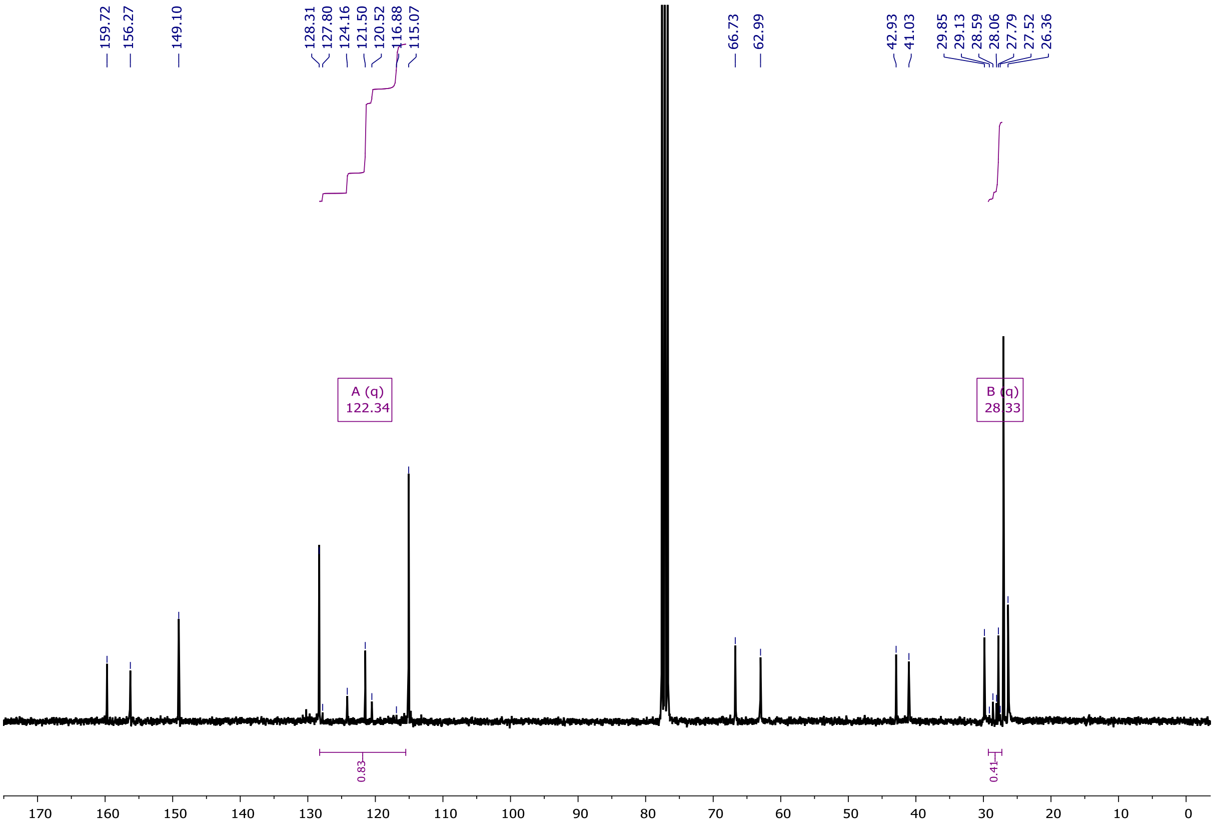

Figure S2. ^{13}C-NMR spectrum of PUR-diazirine crosslinker **3** in $CDCl_3$.

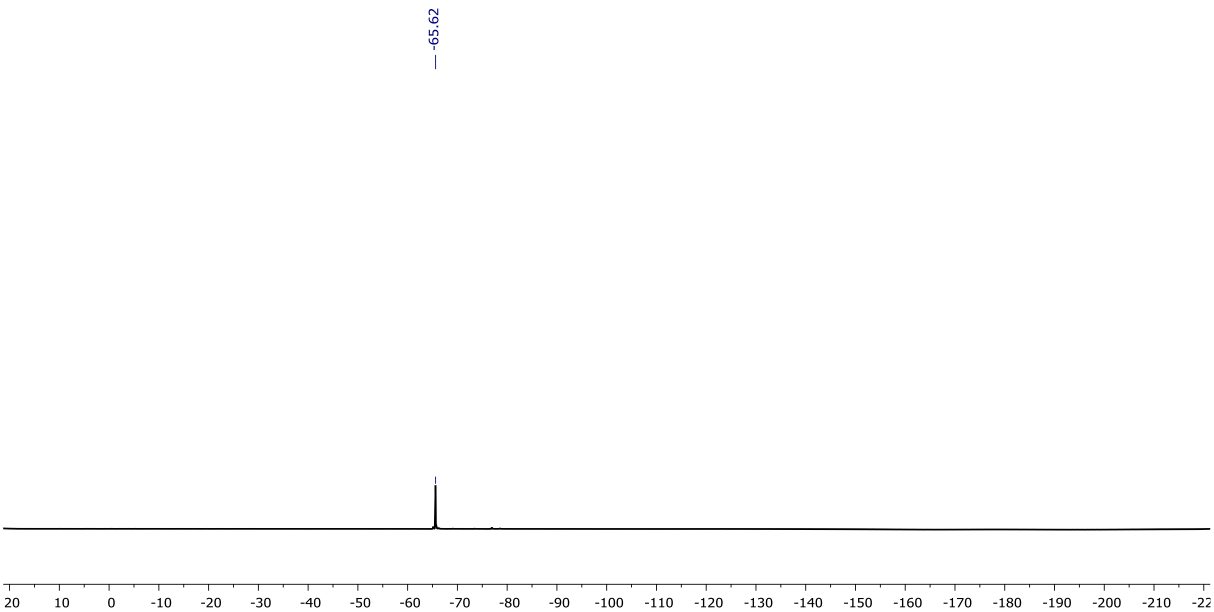

Figure S3. ^{19}F-NMR spectrum of PUR-diazirine crosslinker **3** in $CDCl_3$.

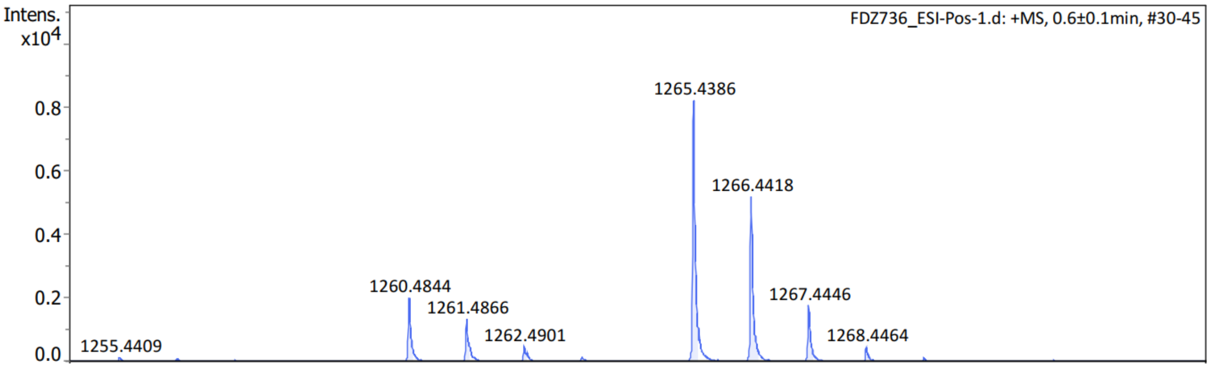

Figure S4. HRMS (ESI) spectrum of PUR-diazirine crosslinker **3**.

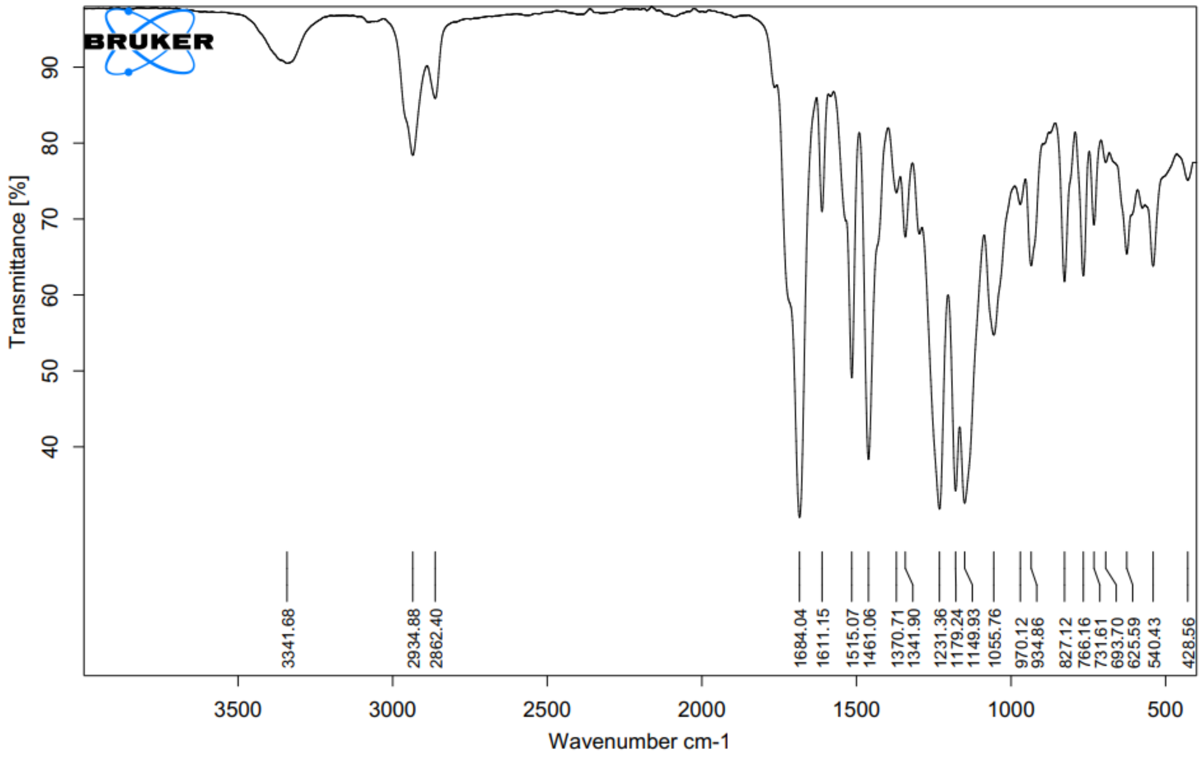

Figure S5. ATR-IR spectrum of PUR-diazirine crosslinker **3**.

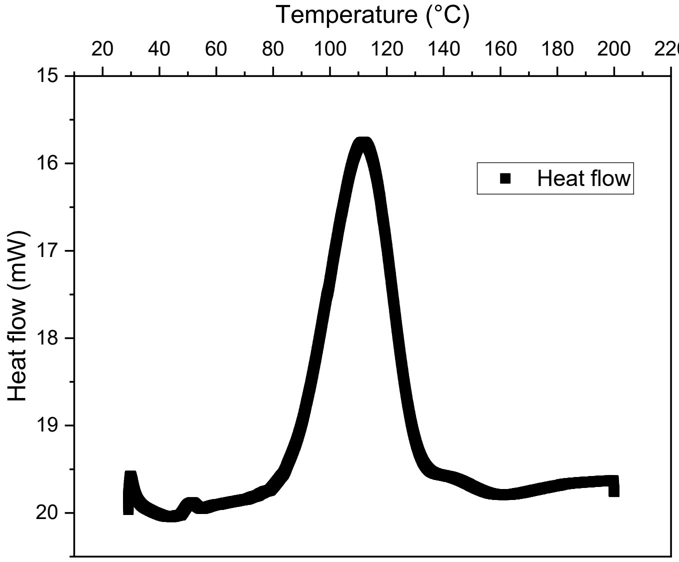

Figure S6. DSC trace of decomposition of PUR-diazirine crosslinker **3** with a 5 °C/min ramp.

4. Characterization of Coatings

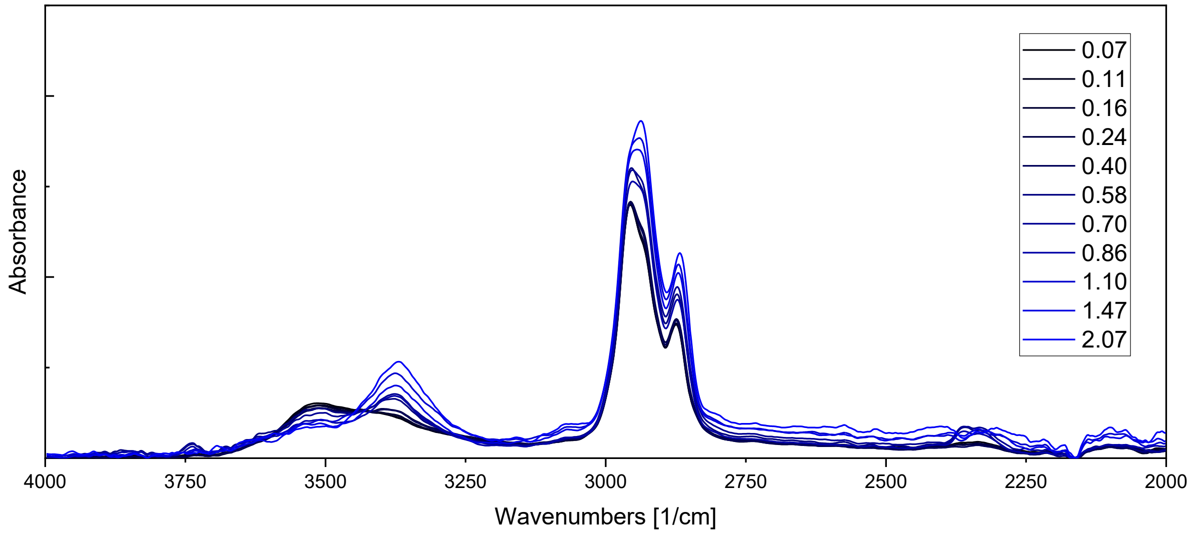

Figure S7. ATR-IR spectrum of APO with increasing amounts of PUR-diazirine crosslinker **3** (mol ratio N_2/OH in legend).

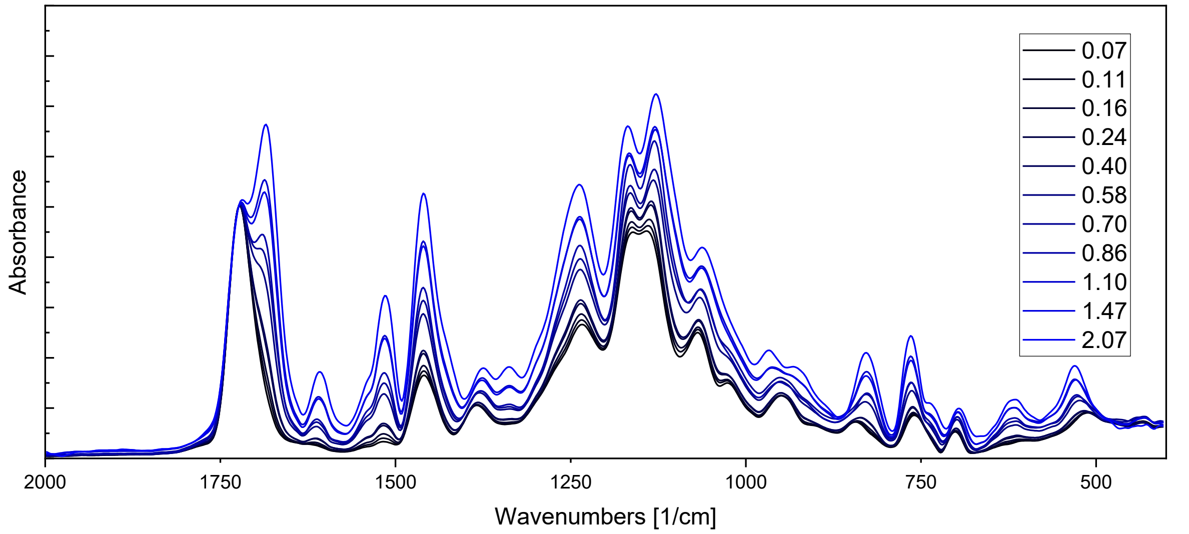

Figure S8. ATR-IR spectrum of APO with increasing amounts of PUR-diazirine crosslinker **3** (mol ratio N_2/OH in legend).

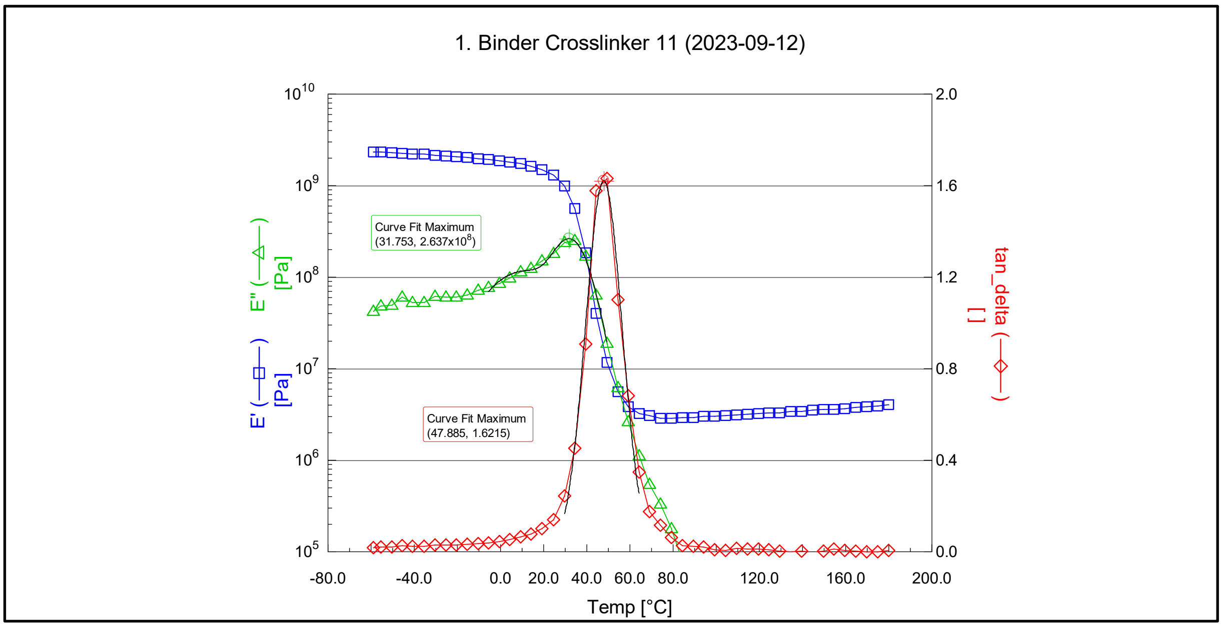

Figure S9. DMTA of PUR/APO coating containing a 1:1 ratio of N_2/OH.

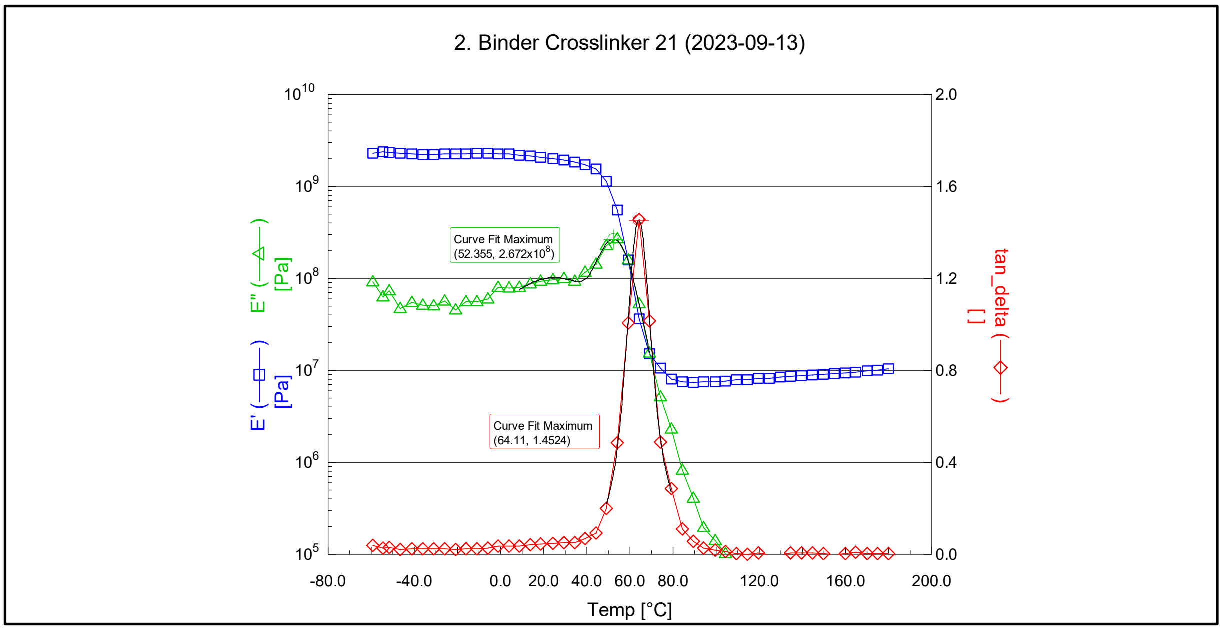

Figure S10. DMTA of PUR/APO coating containing a 2:1 ratio of N_2/OH.

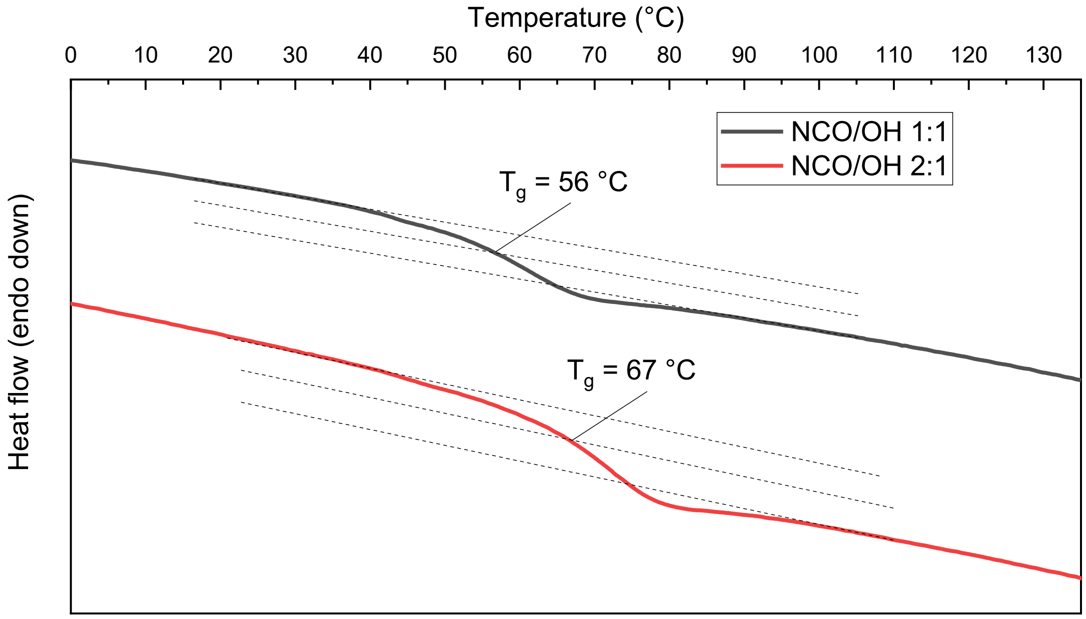

Figure S11. DSC trace of isocyanate/APO coating containing a 1:1 (black) or 2:1 (red) ratio of NCO/OH.

References

1 Bi, L.; Godwin, B.; Baran, M. J.; Nazir, R.; Wulff, J. E. A Cleavable Crosslinking Strategy for Commodity Polymer Functionalization and Generation of Reprocessable Thermosets. *Angew. Chem. Int. Ed.* **2023**, *62*, e2023047.

2 De Zwart, F. J.; Wolzak, L. A.; Laan, P. C. M.; Mathew, S.; Flapper, J.; van den Berg, K. J.; Reek, J. N. H.; de Bruin, B. Thermal/Blue Light Induced Cross-Linking of Acrylic Coatings with Diazo Compounds. *Macromol. Rapid Commun.* **2023**, *44*, 2300380.

3 Van Engelen, A. H. G.; Koenraadt, M. A. A. M.; Van Der Putten, A. J.; Akzo Nobel Coatings International BV. Composition A4. U.S. Patent US8133953B2, 2012.

4 Hermens, J. G. H.; Freese, T.; van den Berg, K. J.; van Gemert, R.; Feringa, B. L. A Coating from Nature. *Sci Adv.* **2020**, *6*, eabe0026.

5 Flory, P. J. Molecular theory of rubber elasticity. *Polym. J.* **1985**, *17*, 1–12.
